# Acoustic Identification of Sentence Accent in Speakers with Dysarthria: Cross-Population Validation and Severity Related Patterns

**DOI:** 10.3390/brainsci11101344

**Published:** 2021-10-13

**Authors:** Viviana Mendoza Ramos, Anja Lowit, Leen Van den Steen, Hector Arturo Kairuz Hernandez-Diaz, Maria Esperanza Hernandez-Diaz Huici, Marc De Bodt, Gwen Van Nuffelen

**Affiliations:** 1 Department of Otorhinolaryngology, Head and Neck Surgery and Communication Disorders, University Hospital of Antwerp, Wilrijkstraat 10, 2650 Edegem, Belgium; Leen.VandenSteen@uza.be (L.V.d.S.); Marc.DeBodt@uza.be (M.D.B.); Gwen.VanNuffelen@uza.be (G.V.N.); 2 Faculty of Medicine and Health Sciences, Antwerp University, Wilrijkstraat 10, 2650 Edegem, Belgium; mariahuici@gmail.com; 3 School of Psychological Sciences and Health, University of Strathclyde, 40 George Street, Glasgow G1 1QE, Scotland, UK; a.lowit@strath.ac.uk; 4 Faculty of Electrical Engineering, Central University Marta Abreu of Las Villas, C. Camajuani km 5.5, Santa Clara 50100, Cuba; akairuz1985@gmail.com; 5 Faculty of Medicine and Social Health Sciences, University of Ghent, De Pintelaan 185, 9000 Gent, Belgium

**Keywords:** sentence accent, dysarthria, acoustic features, prosody

## Abstract

Dysprosody is a hallmark of dysarthria, which can affect the intelligibility and naturalness of speech. This includes sentence accent, which helps to draw listeners’ attention to important information in the message. Although some studies have investigated this feature, we currently lack properly validated automated procedures that can distinguish between subtle performance differences observed across speakers with dysarthria. This study aims for cross-population validation of a set of acoustic features that have previously been shown to correlate with sentence accent. In addition, the impact of dysarthria severity levels on sentence accent production is investigated. Two groups of adults were analysed (Dutch and English speakers). Fifty-eight participants with dysarthria and 30 healthy control participants (HCP) produced sentences with varying accent positions. All speech samples were evaluated perceptually and analysed acoustically with an algorithm that extracts ten meaningful prosodic features and allows a classification between accented and unaccented syllables based on a linear combination of these parameters. The data were statistically analysed using discriminant analysis. Within the Dutch and English dysarthric population, the algorithm correctly identified 82.8 and 91.9% of the accented target syllables, respectively, indicating that the capacity to discriminate between accented and unaccented syllables in a sentence is consistent with perceptual impressions. Moreover, different strategies for accent production across dysarthria severity levels could be demonstrated, which is an important step toward a better understanding of the nature of the deficit and the automatic classification of dysarthria severity using prosodic features.

## 1. Introduction

Prosody forms part of the suprasegmental characteristics of speech and reflects meaningful variations in pitch, loudness, length, and pause across words, phrases, and sentences. Prosodic disturbances are considered a perceptual hallmark of dysarthria, a neurological motor speech disorder [1,2,3]. Common manifestations include a slowed or accelerated speech rate, monopitch and monoloudness, rhythmic disturbances and reduced ability to vary stress and accent [2,3,4,5,6,7,8,9,10]. Stress is a structural, linguistic property of a word that specifies which syllable in the word is more prominent than the others, and it is determined by the language system [11,12,13]. Accentuation is associated with the communicative intention of highlighting important information within utterances (dependent on language behaviour), also known as focus [4,11,12,13,14,15].

Changes in pitch, loudness and/or duration—represented acoustically by changes in fundamental frequency (F0), intensity (I) and duration [4,16]—enable the speaker to make a clear distinction between the more and less important parts of his utterance. Consequently, effective accent or focus placement on an utterance is essential for the efficient conveyance of meaning [17], and disturbances can lead to reductions in intelligibility and naturalness of speech [1,6,8,16,18].

Previous case studies suggest that healthy speakers and speakers with dysarthria rely on different combinations of changes in F0, intensity, and duration to achieve sentence accent [9,10,19,20,21,22,23,24], but more profound insight into these strategies is lacking. Objective analysis and detection of valid acoustic descriptors of sentence accent may increase our understanding and support the clinical assessment of speech in patients with dysarthria [3].

Studies of acoustic correlates of sentence accent have provided valuable insight into this domain [12,25,26,27,28,29]. Acoustic accent production descriptors have been studied in healthy speech [30,31,32,33,34,35,36,37] and in dysarthria [9,10,16,24,38,39]. Currently, there is general agreement in the literature that syllable duration, pitch pattern, and intensity (or sub-band energy) correlate with accentuation [26,40]. These acoustic parameters have been used in systems for automatic accent detection; however, there is a lack of validated automatic analysis techniques to investigate accent production in disordered populations.

A recent study by Mendoza et al. [41] analysed the speech samples of 30 healthy control participants (HCP) and 50 participants with dysarthria, including different aetiologies and severity levels (ranging from mild to severe), who are all native Dutch speakers. The study demonstrated that sentence accent production could be characterised using a set of ten acoustic features. The selected features included not only the traditional values of F0, intensity, and duration measured within the target syllables, but also the differences of these three parameters with the values of their preceding syllable and with the median values of the entire utterance. These acoustic features demonstrated how a speaker manipulated F0, intensity, and duration to accentuate the target syllable within their prosodic capabilities. Furthermore, the combination of these features also allowed a reliable classification between accented and unaccented syllables in healthy and pathological speech. The study demonstrates the value of considering a more comprehensive range of variables and how they interact with each other in investigations of sentence accent production. However, further validation of the set of variables and the developed automatic analysis is necessary across different speaker populations varying in the type and severity of their speech disorders and spoken language.

Consequently, the purpose of this study is twofold. First, it aims to validate the methodology used by Mendoza et al. [41] with a new sample of British English speakers. Although most Germanic languages tend to produce sentence accent similarly [11,12,19,20,42], there might be subtle differences. Therefore, it is important to investigate whether Mendoza et al. [41] feature pool can equally distinguish accented from unaccented syllables in impaired speech in other languages. Second, the study aims to investigate the extent to which a more detailed analysis of accent production has the potential to reflect the severity of dysarthria.

## 2. Materials and Methods

### 2.1. Speech Samples

Samples of adult native speakers of Dutch and English were analysed. For the Dutch samples, 30 HCP and 50 participants with different types of dysarthria (spastic, flaccid, ataxic, hypokinetic, unilateral upper motor neuron (UUMN), mixed) and all severity levels were selected from the ‘Computerized Assessment and Treatment of Rate, Intonation, and Stress’ (CATRIS) corpus [43], which was composed for prosody research. It contains samples from 36 control and 55 speakers with dysarthria and different types of speech tasks. They all reported sufficient visual and auditory abilities to participate in the study. Cognitive skills were not explicitly screened, but all participants demonstrated sufficient abilities to understand and perform the assessment instructions appropriately. For the present study, only speech samples from the focus communicative function were selected, 1 of the 55 participants did not perform the focus task, and other samples from 10 speakers (6 HCP and 4 with dysarthria) were not included due to poor acoustic quality. The group with dysarthria included 31 male and 19 female participants with an age range between 30 and 87 years (mean = 61 years, std = 13 years). The control group included 10 male and 20 female participants with an age range between 18 and 75 years (mean = 40 years, std = 15 years).

For the English samples, eight out of ten speakers with hereditary ataxia and dysarthria first described in Lowit et al. [9] were selected. Two speakers were excluded due to background noise in the audio recordings, making them unsuitable for the automatic analysis. The hearing and vision of all participants were normal or corrected-to-normal, and they had no significant cognitive deficits. The group included 3 male and 5 female participants with an age range between 28 and 72 years (mean = 52 years, std = 16 years).

The dysarthria severity level of all individuals ranged from mild over moderate to severe and was rated with a four-point grading scale (0 = normal; 1 = mild; 2 = moderate; 3 = severe) by three experienced speech and language pathologists (SLPs). Table 1 and Table 2 summarise the selected speakers’ characteristics, based on the severity of dysarthric features and perceptually rated intelligibility.

### 2.2. Speech Production Tasks

The speech material consisted of a comparable set of sentences in both languages based on the standard paradigm to elicit sentence accent, i.e., repetitions of the same sentence with varying accent positions depending on the asked question [9,39,44,45,46]. The questions were structured to elicited new information rather than contrastive focus. For the Dutch speakers, the sample included 3 different sentences. Each sentence was elicited twice, with the focus occurring either in the initial (one case), medial (two cases), or final sentence position (three cases), resulting in six productions per participant, Table 3. A total of 180 and 300 sentences were available for the control and dysarthric group, respectively. For the English sample, speakers produced a set of 10 sentences, in which the focus was either in the initial, medial, or final position (10 cases for each position), resulting in 30 productions per speaker. A total of 240 sentences were included for this group. Table 3 summarises the focus sentences used in each language. In all cases, the participants were instructed to accent the typographically highlighted word.

In the Dutch samples, the perceived accent of each sentence was assessed by three experienced clinicians, who independently marked the accented syllables without knowledge of the target word. If at least two judges had assigned the label ‘accented’ to a particular syllable, that label was retained. For the perceptual analysis of the English speakers, five SLPs judged the samples, deciding whether single or multiple elements were accented and indicating their respective locations [9]. They had the option of selecting a single or multiple accented word. Decisions on which syllable(s) was accented were again made by majority rule. Native speakers with intact hearing completed all the evaluations. Table 4 summarises the number of perceived accented syllables included in each group’s analysis.

### 2.3. Acoustic Analysis

#### 2.3.1. Set of Parameters Used to Detect and Describe Sentence Accent

The acoustic analysis was performed with MatLab software (version 2019b). An automatic algorithm described by Mendoza et al. [41] was used to detect the syllable nuclei and extract fundamental frequency (F0), energy, and duration. F0 values and intensity values were then normalised to make them speaker-independent, i.e., F0 was transposed into semitones (ST) [47], and intensity was normalised with respect to the maximum amplitude value of each sentence. Duration values (D) are reported as absolute values in milliseconds (ms). The algorithm automatically detected syllable nucleus boundaries, energy envelopes, and F0 and plotted them over the spectrogram of each utterance, where they could be visually and auditorily inspected as well as manually corrected if necessary (see [41], for a detailed description of the automatic algorithm). Syllable nucleus boundaries had been manually modified when the algorithm had erroneously marked the boundaries, and corrections of F0 were required in 25 sentences of the total population (3.47%). Subsequently, a total of ten acoustic features were calculated for each syllable nucleus within an utterance (Table 5). Three parameters were inherent to each syllable, four were calculated in comparison with the previously uttered syllable and three others in comparison with the median of the entire sentence (the median was selected because it is a good estimator of central tendency in small samples). These parameters were then used to determine the acoustic differences between perceptually identified accented and unaccented syllables.

#### 2.3.2. Accent Detection in Dysarthric Speech and Description by Severity Levels

The set of independent acoustic features outlined in Table 5 was used as a predictor of accent placement. Discriminant analysis was performed to determine a linear combination of the ten parameters that enables the identification of the following two categories: accented and unaccented syllables [48,49]. The coefficients for the linear equation were calculated based on the group of Dutch speakers with dysarthria (Table 4). The discriminant analysis was performed using the Statistical Package for the Social Sciences (SPSS) software (version 21). The discriminative capacity of the equation was then validated with the English corpus of speakers with ataxic dysarthria, which included a total of 2247 syllables classified as accented or unaccented.

To investigate the strategies used for accent production across the different dysarthria severity levels (DSL), we performed a discriminant analysis using the SPSS software (version 21). For this analysis, the Dutch and English data were merged. The front-end processing used the set of ten acoustic features previously defined in Table 5, the independent variables were used together, and the Wilks’s Lambda criteria was selected [48]. The different severities of dysarthria (mild, moderate, and severe) were analysed separately; the aim was to identify the contribution of each acoustic feature to accent production for each severity level in order to look for possible differences in the accentuation patterns.

## 3. Results

### 3.1. Validation of the Acoustic Features for Accent Detection

The discriminant analysis was initially performed with the samples of the Dutch-speaking population with dysarthria. As a result, the unstandardised discriminant function coefficients were obtained (Table 6). They were used to construct the following actual prediction Equation (1), which was used to classify the new English language cases in this study:

Y = β_1_* ΔF0 + β_2_ * Int + β_3_ * F0 & Int + β_4_ * dF0min + β_5_ * dF0max + … + C (1)

where Y is the discriminant score, β’s are the unstandardised discriminant function coefficients, and C is a constant. Y is the score obtained from the linear combination of the β coefficients (listed in Table 6) multiplied by each discriminant feature. For Y ≥ 0.86, the syllable is classified as accented; for Y < 0.86, the syllable is unaccented. The cut-off value (0.86) is the mean of the two centroids (Table 7), which are the mean value of the discriminant score for a given category (un/accented) of the dependent variable.

**Figure 1:**
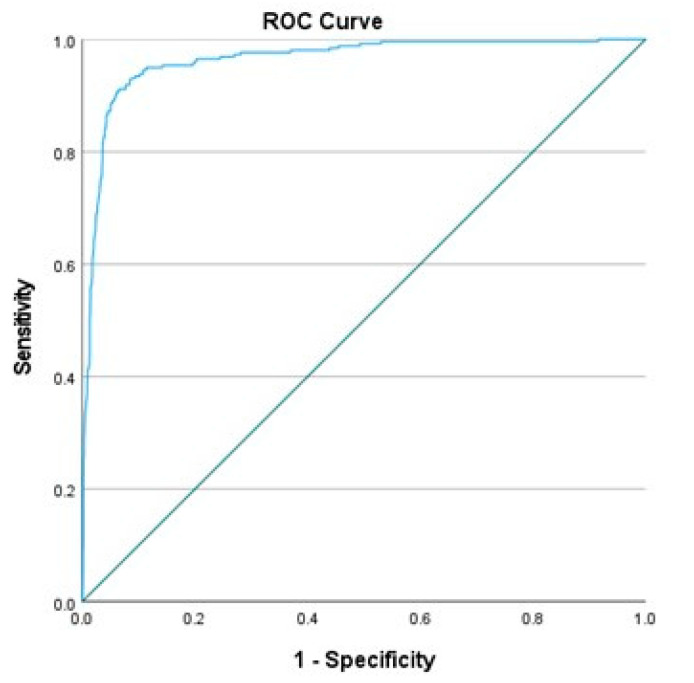
ROC curve representing the discriminatory ability of Equation (1) on the task of detecting un/accented syllables.

The linear combination of the ten acoustic parameters was then applied to classify accented and unaccented syllables in the English sample. As previously reported by Mendoza et al. [41], the results for the Dutch speakers showed a percentage of correct classification of 82.8% for accented syllables and 90.5% for unaccented syllables for the speakers with dysarthria and 87.3 and 96.6% for the control group, respectively. Table 8 shows the confusion matrix with the results (in %) of correct classification for the two categories of the dependent variable (accented versus unaccented syllables) for the newly analysed English corpus, indicating that the approach worked equally well across the two speaker populations. The Receiver Operating Characteristic (ROC) curve was represented in Figure 1; this is a graphical representation of the equation’s performance, representing the true-positive rate against the false-positive rate. The area under the ROC curve (AUC) is a measure of how well the equation can discriminate between the two outputs (un/accented) syllables; for our study, AUC = 0.964, 95% confidence interval: 0.952–0.975, *p* < 0.001.

### 3.2. Impact of Different Dysarthria Severity Levels on Production Patterns for Sentence Accent

The discriminant analysis was applied individually to the different severity levels, showing the standardised canonical discriminant function coefficients per group. The magnitude of these coefficients indicates the relative importance of each independent acoustic feature in predicting the accent. They also allow for a comparison of the parameters measured on different scales (F0, Intensity, Duration). Coefficients with large absolute values correspond to acoustic features with greater discriminating ability. The discriminative coefficients are listed in Table 9.

The values of these coefficients indicated the features predominantly used to produce accent in healthy and dysarthric speech. Variations were observed between the severity levels. For example, the HCP tended to use changes in F0 and intensity (F0 & Int) within the target syllable supported by an increase in F0 in relation to the preceding syllable (dF0max) to produce a detectable accent. The group of speakers with mild dysarthria showed a similar tendency, meaning that they retained control over F0 to highlight important information in the sentence. Then, as the severity progressed, the pattern increasingly deviated from the HCP pattern. The group with moderate dysarthria used the same two main features, although they were not as prominent as in the HCP. In addition, the target syllables were highlighted by means of intensity contrast to the rest of the sentence (IntM). On the other hand, the participants with severe dysarthria only used one of the main features applied by HCP, (dF0max) and supplemented this strategy by manipulating intensity more prominently (Int and IntM). This group of speakers appeared to have less control over F0 but managed to compensate with intensity changes.

## 4. Discussion

### 4.1. Cross-Population Validation of Acoustic Features

This study validated an automatic system that extracts ten specific acoustic features derived from F0, intensity, and duration, used for sentence accent identification across different languages and speaker populations with atypical prosody. The acoustic features were divided into three categories (Table 5), the syllable’s inherent parameters, the parameters of the syllable in contrast with the preceding syllable and the parameters of the syllable in contrast with the entire sentence. This set of features was used in a discriminant function to classify between accented and unaccented syllables, achieving 91.9% of correct classification of accented syllables and 92.2% of correct classification of unaccented syllables for the new population of English speakers affected with ataxic dysarthria. The classification accuracy results are comparable with the results of our previous study for native Dutch speakers (healthy and dysarthric speech) and with other studies of accent detection in healthy speech [30,31,32,33,34,35]. The results suggest that combining the ten acoustic parameters developed by Mendoza et al. [41] has a good capacity to discriminate between accented and unaccented syllables in healthy and speech-impaired speakers of Germanic languages with comparable accentuation patterns, such as English and Dutch.

In clinical practice, this automatic accent detection system could significantly reduce the time required to analyse speech data and provide quantitative information of prosodic parameters that could be useful as diagnostic and outcome measures. This could help clinicians define and implement more precise therapeutic approaches based on the identification of specific compensatory strategies of accent production. In addition, the current system’s focus on within utterance variables may, in the future, allow a move away from structured sentence accent tasks toward more naturalistic speech samples as the basis for analysis, thus providing greater face validity to the information gained from the investigation of both healthy and disordered speech.

This study did not investigate the erroneous classifications in further detail. However, a preliminary inspection of the misclassified syllables showed some utterances where the system detected two accents and the listeners only one. Such cases could indicate specific dysarthric speech deficits such as excess stress where several syllables in an utterance received similar levels of accent as often reported for ataxic dysarthria or the reduced stress characteristic of hypokinetic dysarthria where no syllable, in particular, is highlighted from the rest. In such cases, listeners might have felt compelled to identify a single accent target, leading to the mismatch between perceptual and acoustic analysis results. Further investigation of such utterances and perceptual studies of what prompts a listener to identify a particular word in an utterance as accented may shed more light on these cases in the future. In the meantime, it is important to keep in mind that the so-called errors made by the automatic analysis might not reflect analysis mistakes but additional features of dysarthric speech performance.

### 4.2. Impact of Severity on Accent Production

As the current data show, the ability to accentuate or highlight information within an utterance is not related to the overall severity of the dysarthria, as even the severely affected participants (SAP) managed to place the accent in their sentences successfully. However, limited information is available to date on whether severity influences the acoustic patterns used to signal sentence accent. This could guide more effective intervention approaches for the speakers. Previous research, such as a study by Lowit et al. [9], did not find a strong correlation between dysarthria severity and accent production patterns; however, this study was based on a much-reduced set of features than that applied by Mendoza et al. [41].

The present study shows clear differences in how acoustic prosodic features were manipulated by the different speaker groups. The speakers with mild dysarthria tended to use similar strategies to the control group, i.e., they conveyed accent by making changes to F0 within the target syllable, with a simultaneous increase in the intensity (F0 & Int) and contrast in the frequency between the target syllable and the preceding syllable (dF0max). This result is in line with previous studies that reported F0 as the primary marker for accent perception [50,51,52] and found that speakers with acquired motor speech disorders could use the same pitch patterns as HCP [10,53,54].

The group of moderately affected participants (MAP) appeared to still have control over F0 in the target syllable and in contrast with the preceding syllable, although these features were not used as prominently as in the HCP. As a result, the MAP used additional compensatory strategies to produce accent, i.e., they also applied changes to intensity relative to the rest of the sentence (IntM).

The SAP demonstrated a reduced ability to control F0. However, they compensated for this by using mainly intensity (both within syllables (Int) and compared with the median intensity of the entire sentence (IntM)). This is an interesting observation given that Lowit et al. [38] demonstrated in a perceptual experiment that intensity could be a powerful signal for listeners and used in compensation for pitch. Thus, the speakers with ataxia seem to naturally employ the most effective compensatory feature to counteract their deficit in F0 manipulation.

This objective analysis of accent patterns within different levels of severity contributes to a better understanding of the nature of dysarthric speech and its deviant characteristics. It could also help clinicians determine the remaining acoustical cues for accent production in order to select optimal treatment strategies.

## 5. Limitations and Further Directions

Although the results of this study are promising for the automatic detection of sentence accent in dysarthria within different aetiologies and all severity levels, several topics require further research. The potential of this analysis can go beyond the traditional focus tasks and analyse more natural language production, which is highly important in clinical practice; therefore, this will be considered in future studies. The different patterns of accent production between groups of dysarthria severity levels observed in this study also deserve further investigation. More detailed investigations are required to further validate the generality of these results and to expand their scope. It would be useful to carry out a replication study on a larger population, which would allow for a more robust (statistical) analysis of the results.

As discussed above, there was a mismatch between the un/accented syllables classified by the automatic system and the listeners. Further analysis into listener strategies to identify accented words would be helpful to clarify the extent to which this phenomenon was due to speaker characteristics or measurement errors, which would require further refinement of the acoustic features in order to improve system performance. In addition, the quality of the recordings was not optimal in some samples, further analysis of the degree to which this might have impacted the accuracy of the results would be significant. Future studies could evaluate the algorithm performance in non-Germanic languages. Additionally, an investigation of accentuation patterns within different types of dysarthria might be useful to better understand underlying problems and compensatory strategies. Despite these limitations, the presented method is suitable for future research investigating larger samples of dysarthric speech. It can provide insight into the patients’ motor control processes and support patient-tailored therapeutic interventions—what the problem is and how to compensate for it.

## 6. Conclusions

This cross-population study validated a detailed set of acoustic descriptors related to F0, intensity, and duration (calculated within utterances) used for the automatic detection of sentence accent in dysarthric speech. The discrimination between accented and unaccented syllables using the automatic algorithm was accurate for both populations (Dutch and English speakers with dysarthria).

In addition, the validated acoustic features could adequately describe the strategies used for accent production across different severities. They provided a detailed objective description and a deeper understanding of the strategies or compensatory mechanisms used by speakers with dysarthria to highlight important information in a spoken message.

The clinical significance of this study is threefold: faster automatic detection of accent production, an objective analysis useful as an outcome measure, and support in determining therapeutic strategies.

## Figures and Tables

**Table 1:** Characteristics of the selected Dutch speakers.

			Gender		Dysarthria Severity		
Dysarthria Type	Number of Subjects	Aetiology	F	M	1	2	3
UUMN	2	Stroke-1, TBI-1		2	1	1	
Spastic	2	Stroke-2		2	1		1
Flaccid	7	Encephalopathy-1, Stroke-5, TBI-1		7	5	1	1
Ataxic	2	Neuropathy-1, Stroke-1	1	1	1	1	
Hypokinetic	31	PD-28, Stroke-3	17	14	21	8	2
Mixed	3	ALS-1, Encephalopathy-1, Stroke-1		3	2	1	
Undetermined	3	BT-1, Stroke-2	1	2	3		
Total	50		19	31	34	12	4

Note: M = male; F = female; PD = idiopathic Parkinson’s disease; TBI = Traumatic brain injury; BT = brain tumour; ALS = amyotrophic lateral sclerosis; UUMN = Unilateral upper motor neuron; Dysarthria severity scale: 1 = mild, 2 = moderate, 3 = severe.

**Table 2:** Characteristics of the selected English speakers.

			Gender		Dysarthria Severity		
Dysarthria Type	Number of Subjects	Aetiology	F	M	1	2	3
Ataxic	8	CA-3, FA-3, SCA6-1, SCA8-1	5	3	2	4	2

Note: M = male; F = female; CA = cerebellar ataxia of undefined type; SCA = spinocerebellar ataxia; FA = Friedreich’s ataxia; Dysarthria severity scale: 1 = mild, 2 = moderate, 3 = severe.

**Table 3:** Focus sentences used in each experiment.

Dutch Task	English Task
Ze **wil** geen **telefoon** meer krijgen. *She does not want to get any more calls*.	The **gardener** grew **roses** in **London**.
**Luc** werkt in het **ziekenhuis**. *Luke works at the hospital.*	The **minister** has a **nanny** from **Norway**.
Misschien heeft **Piet vakantie**. *Maybe Pete is on holiday.*	The **model** wrote her **memoirs** in **Lima**.
	The **diva** made a **movie** in **Venice**.
	The **lawyer** met the **model** in **London**.
	The **widow** bought a **villa** in **Ealing**.
	The **neighbour** plays **melodies** on her **mandolin**.
	The **milliner** got a **memo** from **Melanie**.
	The **murderer** met his **lover** in **Limerick**.

Note. English translations in italics for the sentences in Dutch.

**Table 4:** Perceptually detected accented and unaccented syllables for each group of speakers.

Syllables	Speaker Population		
	Control	Dysarthric Dutch	Dysarthric English
Accented	197	338	259
Unaccented	1160	1891	1988
Total	1357	2229	2247

**Table 5:** Set of parameters derived from F0, Duration, and Intensity used in this study [41].

**Parameters inherent to the syllable**
**ΔF0**—the difference between the initial and the final value of F0 within the syllable nucleus in semitones
**Int**—maximum intensity of the syllable nucleus, relative to the overall utterance amplitude envelope
**F0 & Int**—the interaction of F0max (the maximum F0 value within the syllable nucleus) minus F0min (the minimum F0 value within the syllable nucleus) multiplied by Int
**Parameters in comparison with the preceding syllable**
**dF0max**—the difference between the F0max of each syllable with that of the preceding one
**dF0min**—the difference between the F0min of each syllable with that of the preceding one
**dInt**—the difference between Int of each syllable and that of the preceding one
**dDrange**—the difference between the duration of each syllable and that of the preceding one (dD) normalised to the range of all dD values in the sentenceNote. For initial syllables, the second syllable was used to calculate the difference
**Parameters in comparison with the utterance**
**F0maxM**—the difference between F0max and the median F0 of the utterance
**IntM**—the difference between Int and the median Int of the utterance
**DM**—the normalised duration (D, associated with the time length of the syllable nucleus) with respect to the median of all the D values in the sentence

**Table 6:** Canonical Discriminant Function Coefficients.

Parameters	Function
ΔF0	β_1_ = 0.043
Int	β_2_ = 0.605
F0 & Int	β_3_ = 0.119
dF0min	β_4_ = −0.042
dF0max	β_5_ = −0.119
dInt	β_6_ = 0.192
dDrange	β_7_ = 0.336
F0maxM	β_8_ = 0.041
DM	β_9_ = −0.378
IntM	β_10_ = 1.095
Constant	C = −0.595

Note: Unstandardized coefficients.

**Table 7:** Functions at Group Centroids.

Category	Function
Accented	2.094
Unaccented	−0.374

Note: Unstandardised canonical discriminant functions evaluated at group means.

**Table 8:** Confusion matrix for the classification results of the English speakers with dysarthria.

	Predicted Group Membership	
Target Syllables	Accented	Unaccented
Accented	91.9%	8.1%
Unaccented	7.8%	92.2%

**Table 9:** Standardised canonical discriminant function coefficients per group of dysarthria severity levels (mixed groups of English and Dutch speakers).

	Severity Levels			
	Control (30)	Mild (36)	Moderate (16)	Severe (6)
**Parameters** **Inherent to the syllable**				
ΔF0	0.261	0.200	0.184	0.060
Int	0.015	0.104	0.226	**0.240**
F0 & Int	**0.420**	**0.436**	**0.241**	0.175
**In comparison with the preceding syllable**				
dF0min	−0.175	−0.193	−0.200	−0.158
dF0max	**−0.367**	**−0.337**	**−0.271**	**−0.230**
dInt	0.029	−0.046	−0.007	−0.133
dDrange	−0.080	0.157	0.057	0.054
**In comparison with the sentence**				
F0maxM	0.239	0.146	0.113	−0.015
DM	−0.059	−0.263	−0.186	−0.076
IntM	0.212	0.168	**0.435**	**0.568**

## Data Availability

The data that support the findings of this study are available from the corresponding author upon reasonable request.
